# Epidemiology of influenza in Ghana, 2011 to 2019

**DOI:** 10.1371/journal.pgph.0001104

**Published:** 2022-12-09

**Authors:** Ivy Asantewaa Asante, Anne T. Fox, Eric Behene, Yaw Awuku-Larbi, Erasmus Nikoi Kotey, Stephen Nyarko, Richard Asomadu Obeng, Augustina Arjarquah, Gifty Mawuli, Vanessa Magnusen, Naiki Puplampu Attram, Shirley Nimo-Paintsil, Franklin Asiedu-Bekoe, Dennis Odai Laryea, Obed Bangdome Ofori, Edward Owusu Nyarko, Daniel Lartei Mingle, William Asiedu, Andrew Letizia, Terrel Sanders, William Kwabena Ampofo

**Affiliations:** 1 The Virology Department, Noguchi Memorial Institute for Medical Research, University of Ghana, Accra, Ghana; 2 U.S. Naval Medical Research Unit-No. 3, Ghana Detachment, Accra, Ghana; 3 Public Health Division, Ghana Health Service, Accra, Ghana; 4 Disease Surveillance Department, Ghana Health Service, Accra, Ghana; 5 Public Health Division, 37 Military Hospital, Ghana Armed Forces, Accra, Ghana; University Singapore Saw Swee Hock School of Public Health, SINGAPORE

## Abstract

Influenza virus is an important contributor to acute respiratory illnesses and is estimated to cause up to 650,000 respiratory deaths each year. Ghana recorded influenza viruses as far back as 1918 when the Spanish influenza pandemic led to the death of >100,000 people in a population of 4 million at the time. An outbreak of highly pathogenic avian influenza A(H5N1) among poultry in Ghana in 2007, led to the establishment of virological surveillance for influenza-like illness (ILI) by the Noguchi Memorial Institute for Medical Research (NMIMR). This surveillance system, supported by the U.S. Naval Medical Research Unit-No. 3 (NAMRU-3) and the Ghana Health Service (GHS), monitors circulating influenza strains and activity to better understand the epidemiology of influenza in Ghana. We present here the results of this surveillance system from 2011 to 2019. As part of the Integrated Disease Surveillance and Response (IDSR) system of the GHS under the Ministry of Health (MOH), oropharyngeal and nasopharyngeal swabs were collected from patients who met a modified World Health Organization (WHO) case definition for ILI or severe acute respiratory illness (SARI) through a sentinel surveillance system in the country. Samples were transported to the National Influenza Centre (NIC) at the NMIMR and tested for influenza virus using protocols defined by the United States Centers for Disease Control and Prevention (CDC). Selected isolates were sent to the WHO collaborating centre in the United Kingdom for further antigenic characterization. From 2011 to 2019, the NIC tested a total of 21,747 ILI samples and 3,429 SARI samples. Influenza positivity rates were highest in the 5–14 year old group for both ILI (20.8%) and SARI (23.8%). Compared to females, more males were seen at the health facilities for ILI and SARI symptoms with a statistically significant difference in influenza positive ILI (15% vs 13.2%, p <0.001). In terms of absolute numbers, more cases were seen at the health centres during the wet seasons (April to October) compared to the dry seasons (November to March) in Ghana. This study presents 9 years of surveillance data from outpatient and inpatient setting on influenza activity as well as the influenza A subtypes and B lineages that drive the activity. This presents useful information for influenza vaccine selection and administration. Ghana’s unique influenza activity patterns also present a challenge in predicting when an outbreak could occur.

## Background

Globally, acute respiratory infections (ARIs) are a leading cause of infectious disease mortality with an estimated 2.5 million deaths in 2016 [1, 2]. Influenza, an important contributor to ARIs, is now estimated to result in 3–5 million severe illnesses causing up to 650,000 respiratory deaths in seasonal epidemics annually [3]. Various studies have shown that mortality and morbidity associated with influenza varies by age, chronic disease status, and influenza virus type and subtype [4, 5].

The earliest reports of influenza in Ghana date back to the influenza pandemic of 1918–1919, which is described as the worst short-term demographic disaster in the history of Ghana [6]. The disease was likely introduced via shipping routes along the southern coast and spread overland throughout the territory then known as the Gold Coast [6]. Mortality rates varied regionally and, to some extent, by occupation, but the epidemic killed at least 100,000 people in less than six months [6]. Limited influenza data is available from Africa for multiple decades after this pandemic. Subsequent serological investigations of influenza-like illness (ILI) in Ghana demonstrated the presence of influenza A2/Hong Kong/1/68 and influenza A H3N2, in 1973 and 1996, respectively [7, 8].

An outbreak of highly pathogenic influenza A H5N1 among poultry in 2007 and the paucity of influenza data in Ghana, led to initiation of a national surveillance system for ILI by the Noguchi Memorial Institute for Medical Research (NMIMR), with support from the Naval Medical Research Unit-No. 3 (NAMRU-3) and the Ghana Health Service (GHS). This surveillance system aimed to obtain current information about influenza activity, monitor circulating influenza strains, and better understand the epidemiology of influenza in Ghana.

An analysis of data gathered from 2,810 Ghanaian children with ILI between 2008 and 2010, demonstrated slightly higher influenza activity during the rainy season from April to October and an influenza A(H1N1)pdm09 predominance [9]. A separate study of 1,063 children (21% with SARI and 4% with influenza) also demonstrated increased frequency in the rainy season [10]. Influenza A(H1N1)pdm09 emerged in the human population in 2009 and caused the first pandemic of the 21^st^ century. Approximately 80 million cases and 18,500 confirmed deaths were reported globally [11, 12]. However, only 12% of the reported deaths were from Southeast Asia and Africa, which account for almost 40% of the world’s population [13]. Modelling studies estimate that the mortality from the 2009 pandemic was significantly higher than the confirmed numbers with almost 30% of deaths occurring in Africa [11]. Three years later, the World Health Organization (WHO) noted the continued lack of data from sub-Saharan Africa and again called for improved surveillance to understand the impact of ARI in sub-Saharan Africa [14]. The 2009 influenza pandemic, as well as the highly pathogenic avian influenza virus pandemic in 2004, highlighted the need for improved global influenza surveillance especially in lower socio-demographic index (SDI) regions. In addition, robust influenza surveillance programs support the early identification of other respiratory pathogens with epidemic and pandemic potential.

Influenza often manifests with non-specific symptoms presenting a challenge for surveillance, diagnosis, and estimation of disease burden. In addition to the global health effects of influenza on the public health system in Ghana, influenza also remained a high priority respiratory pathogen of U.S. military relevance; respiratory surveillance activities were essential to better understand the transmission dynamics, epidemiologic trends, and emergence of new strains or circulating variations to guide military force health protection (FHP) measures. This study systematically collected data on influenza disease at the population level, evaluated symptomatology, and described the epidemiology of influenza virus as an aetiology for ILI and severe acute respiratory infection (SARI) in Ghana from 2011–2019. Such analysis presents Ghana with an opportunity to decide on influenza vaccine selection and administration which are not currently part of routine immunization in Ghana.

## Methods

### Study sites

The National Influenza Center (NIC) at NMIMR monitors the circulation of influenza viruses in Ghana and provides information to the WHO Global Influenza Surveillance and Response System (GISRS). As part of the Integrated Disease Surveillance and Response (IDSR) system of the GHS and in collaboration with NAMRU-3, 29 sentinel sites were established for influenza virus surveillance in Ghana [15]. The sites, located across the ten regions of Ghana, included eighteen GHS facilities (regional hospitals, municipal hospitals, district hospitals, polyclinics, and health centers) and eleven military medical reception stations (MRS).

**Fig 1:**
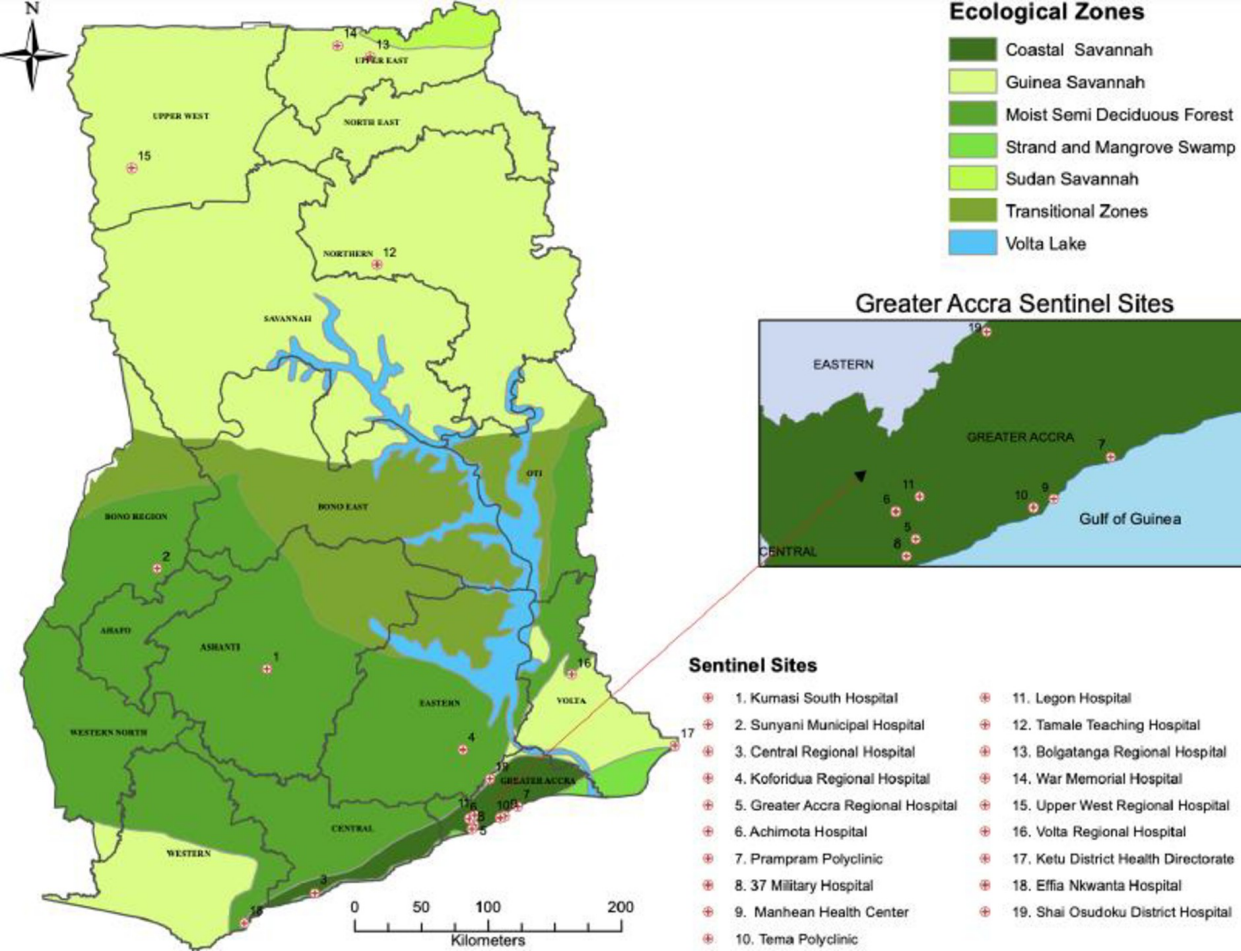
Map of Ghana showing selected influenza sentinel sites located throughout the country. Also shown are the ecological variations of the country. Southern Ghana is green with semi-arid conditions, while Northern Ghana is dry and hot. Southern Ghana is densely populated (Accra-2,291,352) while population density in Northern Ghana is low (562,919-Tamale, Northern Region). Note: Only 19/31 sentinel sites are identified. Those located on Ghana Armed Forces military sites, with the exception of 37 Military Hospital, are not depicted for security reasons. The map was obtained from Centre for Remote Sensing and Geographical Information Services (CERSGIS). This was created using ESRI ArcMap version 10.8 with the boundaries obtained from Ghana Statistical Service (GSS).

The NIC began conducting surveillance for ILI and SARI in 2011 and 2014, respectively. The geographic distribution of the surveillance sites within Ghana (See Fig 1) provides a cross section of climate variation, ecologic zones, population density, and health services availability [16]. The climate varies among 16 regions of Ghana with dry and rainy seasons, depending on distance from the coast [17, 18]. Climate at the coast varies from warm and semi-arid in the east to hot and humid in the west while the north is very hot and arid [17]. The northern region has one rainy season from May to September while the rest of the country has two distinct rainy periods from April to June and September to November [18]. Overall, the wet season in Ghana is known to be from April to October, while the dry season is from November to March. Average rainfall varies from 31 inches in the southeast to 40 inches in the north to 80 inches in the southwest [17]. Population is greatest in Accra, the national capital (4.9 million), and Ashanti (5.8 million) regions, and is lowest in the upper northern regions (2 million) [19].

### Case definitions

The surveillance system utilized a modified WHO case definition for ILI and SARI, per the protocol for human influenza surveillance for Ghana [20]. A person with ILI/SARI was defined as someone with a measured fever of ≥ 38°C/ history of fever and cough and/or other respiratory symptom with onset within the prior 10 days and requiring hospitalization for SARI Demographic details and other symptoms including shortness of breath, headache, myalgia and coryza were also documented.

### Sample collection

Nasopharyngeal and/or oropharyngeal swabs were collected from patients who met the case definition for ILI or SARI based on National Influenza Surveillance guidelines [20]. After collection, the sample swab(s) were placed in either viral transport medium (VTM) or a universal transport medium (UTM). The specimens were stored at 2–8°C at the collection point, transported in a triple packaging system to the NIC within 72 hours of collection, and processed within 72 hours of receipt. The cold chain was maintained throughout the storage and transport processes. Each week, we expected a minimum of 5 ILI and all SARI samples to be shipped from each sentinel site to the NIC. A minimum 5 ILI samples regardless of age and clinical condition seen at the facility were conveniently sampled and shipped to the NIC. For the SARI cases, all patient who met the case definition were sampled and these were shipped to the NIC. All samples were shipped within 72-hours regardless of the day collected. Samples were collected throughout the week excluding weekends.

### Sample processing and virus detection

At the NIC, viral RNA was extracted from 140μl of each specimen using the QIAamp viral RNA mini kit (Qiagen, Germany) according to the manufacturer’s protocol. All purified RNA extracts were first tested by one step real time RT-PCR (rRT-PCR), using the AgPath-ID One-Step RT-PCR Kit (Applied Biosystems, USA) or SuperScript III Platinum One-Step quantitative RT-PCR kit (Invitrogen, USA). Viral antigen detection, characterization, and subtyping was conducted using specific primers and probes described by the United States Centers for Disease Control and Prevention (U.S. CDC) for seasonal influenza A subtypes and B lineages (A[H1], A[H3], A[H1]pdm09, B Yamagata and B Victoria) [21, 22].

Final results were interpreted and recorded as cycle threshold values (CT). The controls and samples were considered positive when CT values were ≤ 37. Any specimen positive for influenza A or B virus that could not be sub-typed was sent to the WHO Influenza Collaborating Centre in London for further antigenic characterization. Influenza virus isolation was attempted for all influenza positive cases using Madin-Darby Canine kidney (MDCK) cell cultures.

### Epidemiological analysis

Frequency and percentage were used to describe the categorical variables. The number of influenza subtypes and positivity rate was described using bar chart and line graph respectively. Association between influenza status with gender, age, season, year samples collected, and clinical symptoms were determined using Chi-square test. A univariate logistic regression with age as exposure variable was regressed on influenza status (outcome variable) in which a pairwise comparison for age was obtained using the Bonferroni method. Significance level for the Bonferroni method was obtained by dividing 0.05 by the number of pairwise comparisons. Associations between influenza positivity rates and clinical symptoms were calculated using logistic regression to estimate odds ratios with its 95% confidence intervals. Statistical analysis was done using STATA version 13 (StataCorp Texas, USA) and graphs were generated using Graph pad prism version 9.0.0.

Moving epidemic method (MEM), a sequential analysis that calculates intensity thresholds, was used to establish seasonality of influenza using weekly influenza positivity rates from the 2011 to 2019 seasonal data. The surveillance season was from Week 1 to 52 as per the ISO week date standard (ISO-8601) used by the WHO Global influenza programme in their reporting. The seasonality which includes the epidemic and intensity thresholds, average start and end of the influenza season epidemics with their confidence intervals were obtained from the analysis of the MEM web application (R version 4.2.1). Influenza thresholds of activity were defined as below seasonal threshold, low activity, moderate activity, high activity, and very high activity. The 40^th^, 90^th^ and 97.5^th^ percentiles were established using historical data to calculate activity thresholds using MEM.

### Ethics statement

This epidemiologic activity was approved by the Noguchi Memorial Institute for Medical Research Institutional Review Board and the Naval Medical Research Center Institutional Review Board (# NAMRU3-PJT-21-01) and Office of Research Administration as public health surveillance. The Ghana Health Service also concurred that this protocol is exempt from ethical considerations including informed consent process because it falls under public health surveillance. The sentinel sites for routine national influenza virus surveillance in Ghana were set up in collaboration with the Ghana Health Service as well as the Ghana Armed Forces as part of the Integrated Disease Surveillance and Response (IDSR) system of the Ghana Health Service, which is the health delivery division within the Ministry of Health. All procedures were performed according to relevant guidelines and regulations. No administrative permissions were required to access this data. Data is kept on a password-protected computer and backed up daily. Data was anonymized before use. Only laboratory identities were used during data analysis.

## Results

### Demographics and influenza-associated ILI

**Fig 2:**
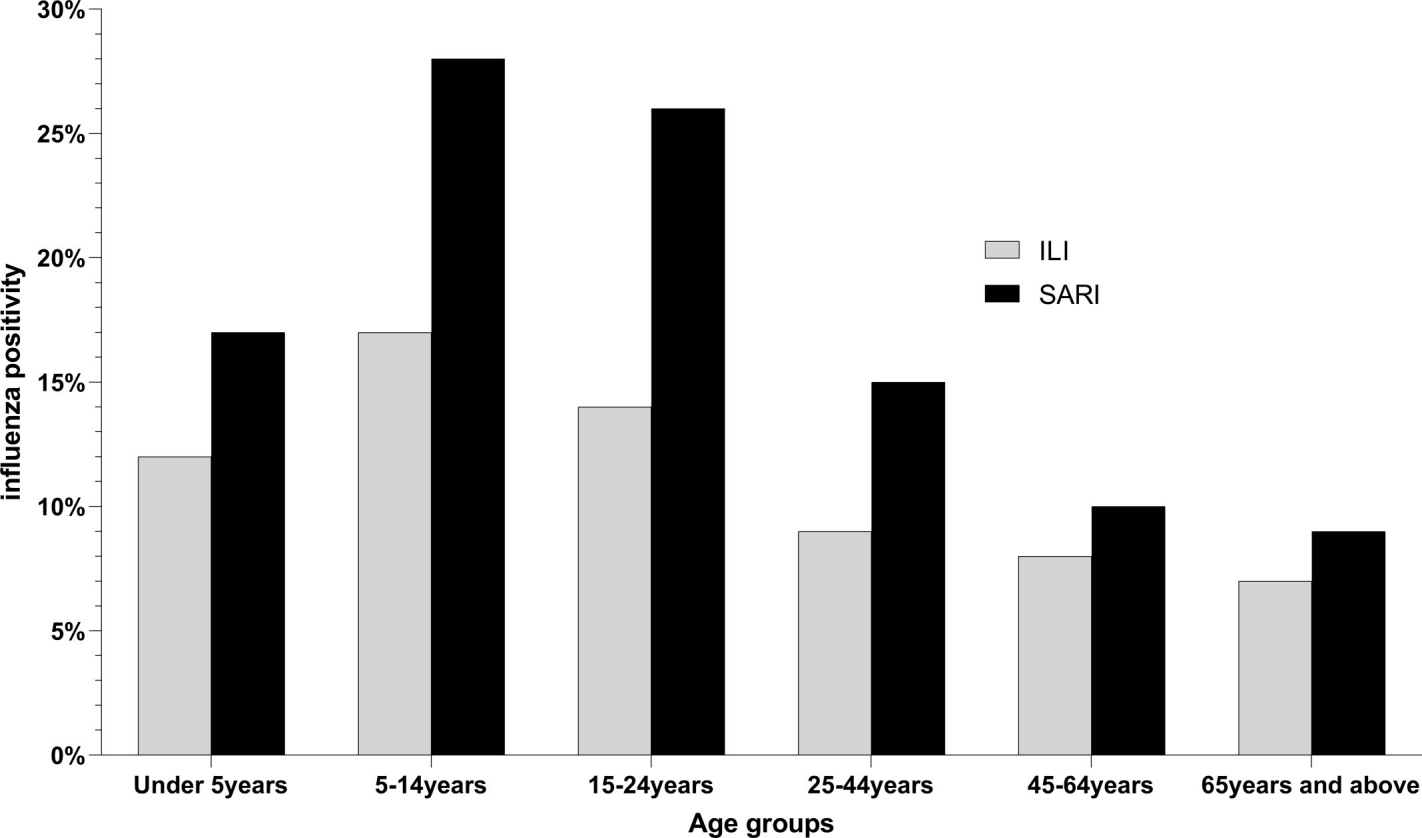
Distribution of influenza positive cases among age groups in Ghana from 2011 to 2019. Influenza like illness (ILI) cases are shown in grey while severe acute respiratory illness (SARI) cases are shown in black.

During the surveillance period, the NIC received and processed a total of 25,176 samples out of which 21,747 (86%) were from ILI and 3,429 (14%) from SARI cases (See Table 1). Of the ILI samples processed, 3,051 (14%) were positive for influenza. Children aged 5–14 years recorded the highest influenza positivity rate (21%, 702/3376) (See Table 1). The influenza positivity rate declined with each subsequent age bracket (See Fig 2). Influenza positivity rates varied significantly between age groups for both ILI and SARI (S1 Table). Of the recorded ILI cases, 53% of the samples were from female patients. There was a statistically significant difference in influenza positivity rate between genders (p<0.001) (See Table 1).

**Table 1:** Demographic characteristics of influenza-like illness (ILI) and severe acute respiratory illness (SARI) cases in Ghana from 2011 to 2019.

	ILI^a^ January 2011-Dec.2019 (n = 21,747)			SARI^b^ January 2011-Dec.2019 (n = 3,429)		
	# Tested	# Influenza Positive (%)	p-value^c^	# Tested	# Influenza Positive (%)	p-value^c^
**Age**			<0.001			<0.001
Under 5years	5584	787 (14.1)		1432	219 (15.3)	
5-14years	3376	702 (20.8)		450	107 (23.8)	
15-24years	3512	615 (17.5)		360	86 (13.2)	
25-44years	5715	632 (11.1)		555	73(13.2)	
45-64years	2453	202 (8.2)		399	34 (8.5)	
≥65years	799	66 (8.3)		196	13(6.6)	
Age Missing	308	47 (15.3)		37	3 (8.1)	
**Gender**			<0.001			0.454
Male	10137	1522 (15.0)		1860	302 (16.2)	
Female	11572	1526 (13.2)		1567	233 (14.9)	
Gender Missing	38	3 (7.9)		2	0 (0.0)	
**Seasons**			<0.001			0.050
Wet	12874	1693 (13.2)		2060	301(14.6)	
Dry	8873	1358(15.3)		1369	234 (17.1)	
**Year**			<0.001			<0.001
2011	2142	351 (16.4)				
2012	1673	170 (10.2)				
2013	1239	65 (5.3)				
2014	1085	93 (8.6)		177	9 (5.1)	
2015	826	115 (13.9)		202	24 (11.9)	
2016	2020	312 (15.5)		417	42 (10.1)	
2017	4105	352 (8.6)		946	116 (12.3)	
2018	4302	620 (14.1)		810	138 (17.0)	
2019	4355	973(22.3)		877	206(23.5)	

^a^ILI: measured fever (≥38°C) with cough, symptom onset within the prior 10 days

^b^SARI: measured fever or history of fever with cough, symptom onset within the prior 10 days and hospitalization

^c^ p-value was obtained using Pearson Chi-square test

^d^ Wet season occurs from April to October and dry season from November to March

**Fig 3:**
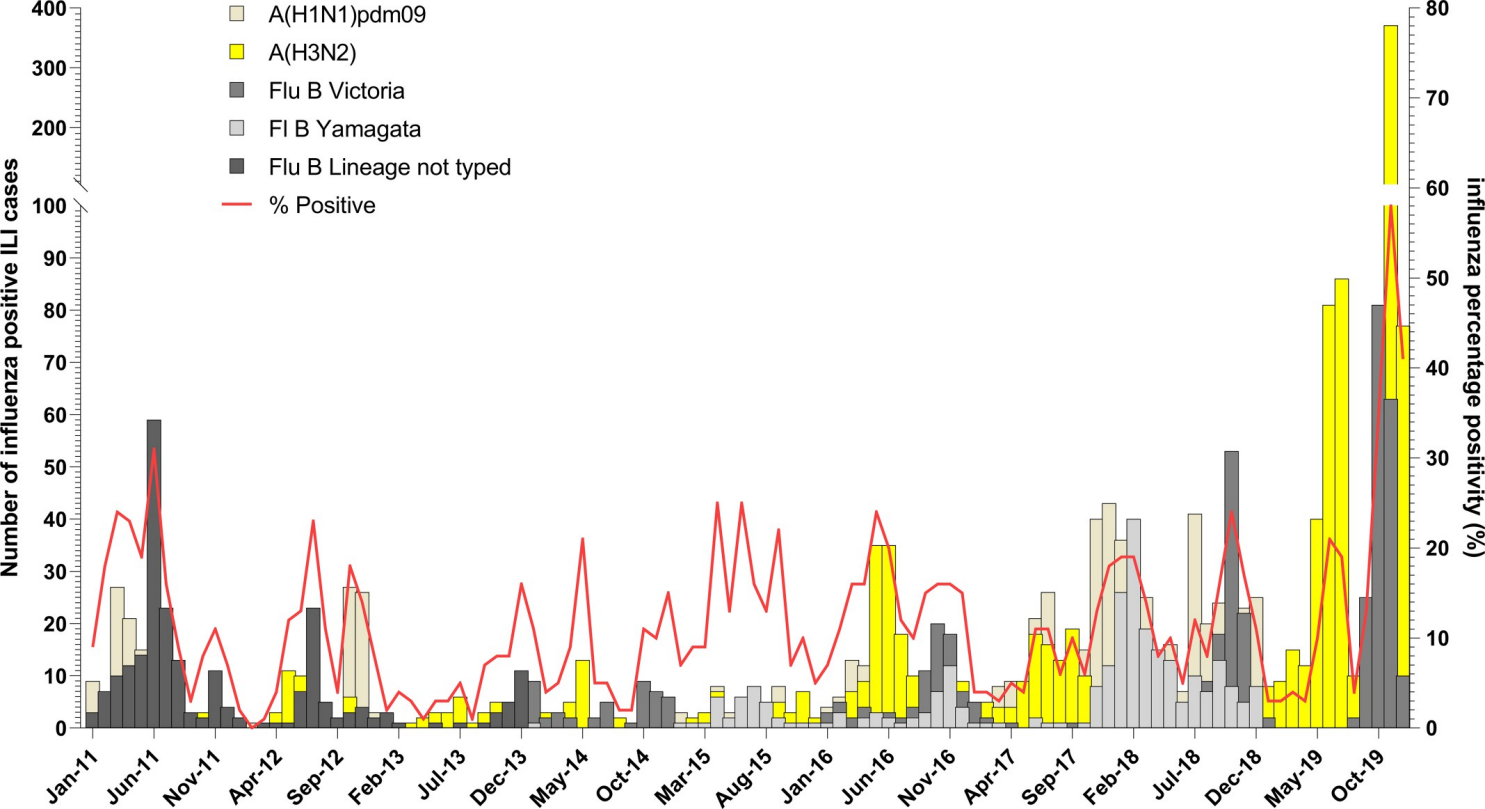
Distribution of influenza A subtypes and B lineages by months in Ghana from 2011 to 2019. Influenza positivity rate and the number of influenza A subtypes and B lineages was plotted against months for ILI.

**Fig 4:**
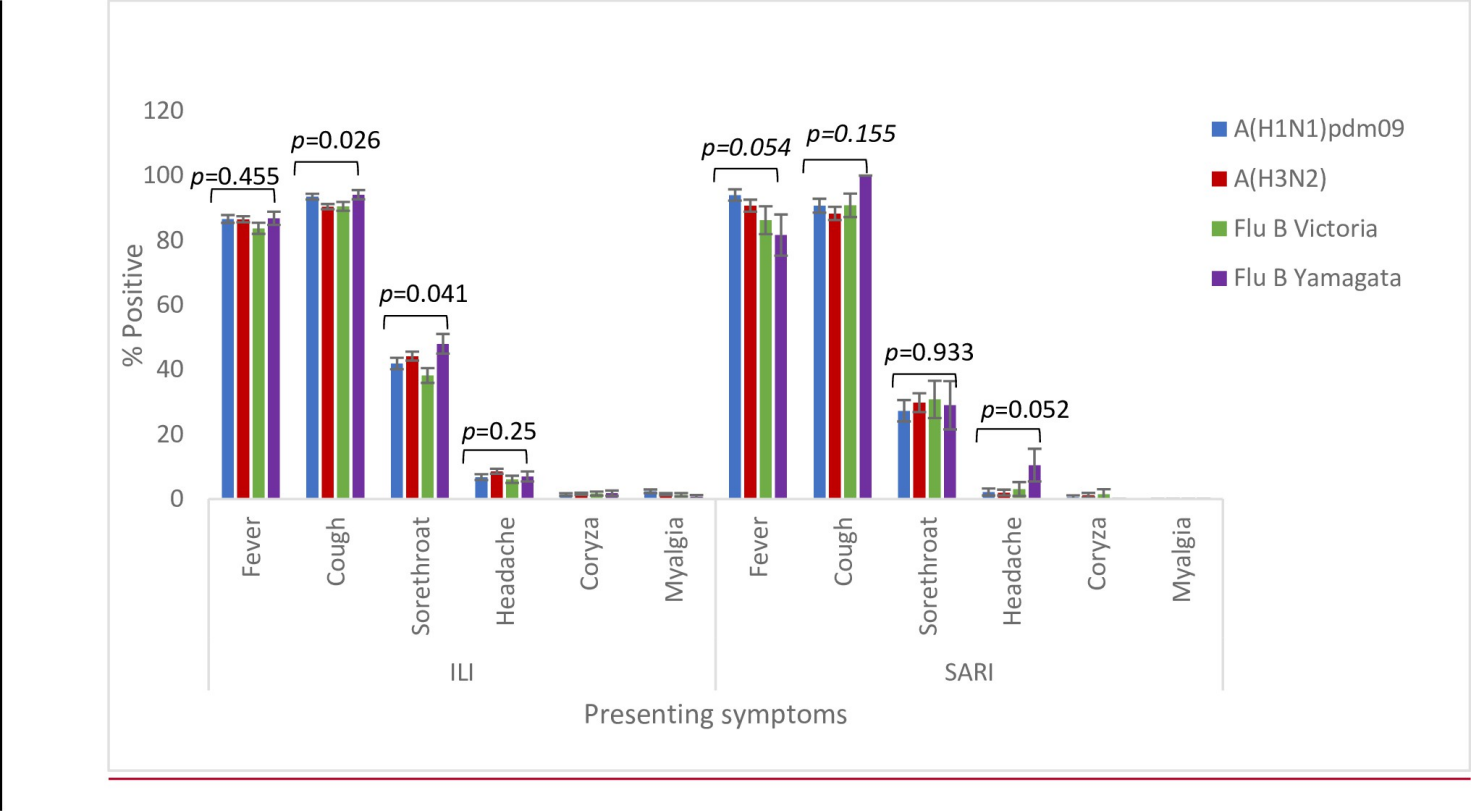
Symptom distribution of ILI and SARI cases based on influenza sub-types in Ghana. *Pearson Chi-square test was used to obtain the p-values.

More ILI samples were processed in the wet season than in the dry season (12,874; 59.2% vs. 8,873; 40.8%). Influenza associated ILI positivity rate recorded during the two seasons was significantly different (p<0.001) with 13% and 15% in the wet and dry seasons, respectively (See Table 1). Although influenza A (H3N2) was commonly detected in both the wet and dry season compared to pandemic A(H1N1)pdm09, the variation in the positivity rates were not statistically significant. However, for influenza B the positivity rates for Yamagata and Victoria varied significantly in both dry and wet seasons. Interestingly, in 2019, there was a predominance of influenza A (H3N2) and influenza B Victoria lineage detected (See Fig 3). There were no significant associations between any of the symptoms and a specific influenza subtype or lineage for ILI cases except cough and sore throat (See Fig 4). See S2 Table for associations between symptoms and influenza positivity rate.

### Influenza associated SARI

**Fig 5:**
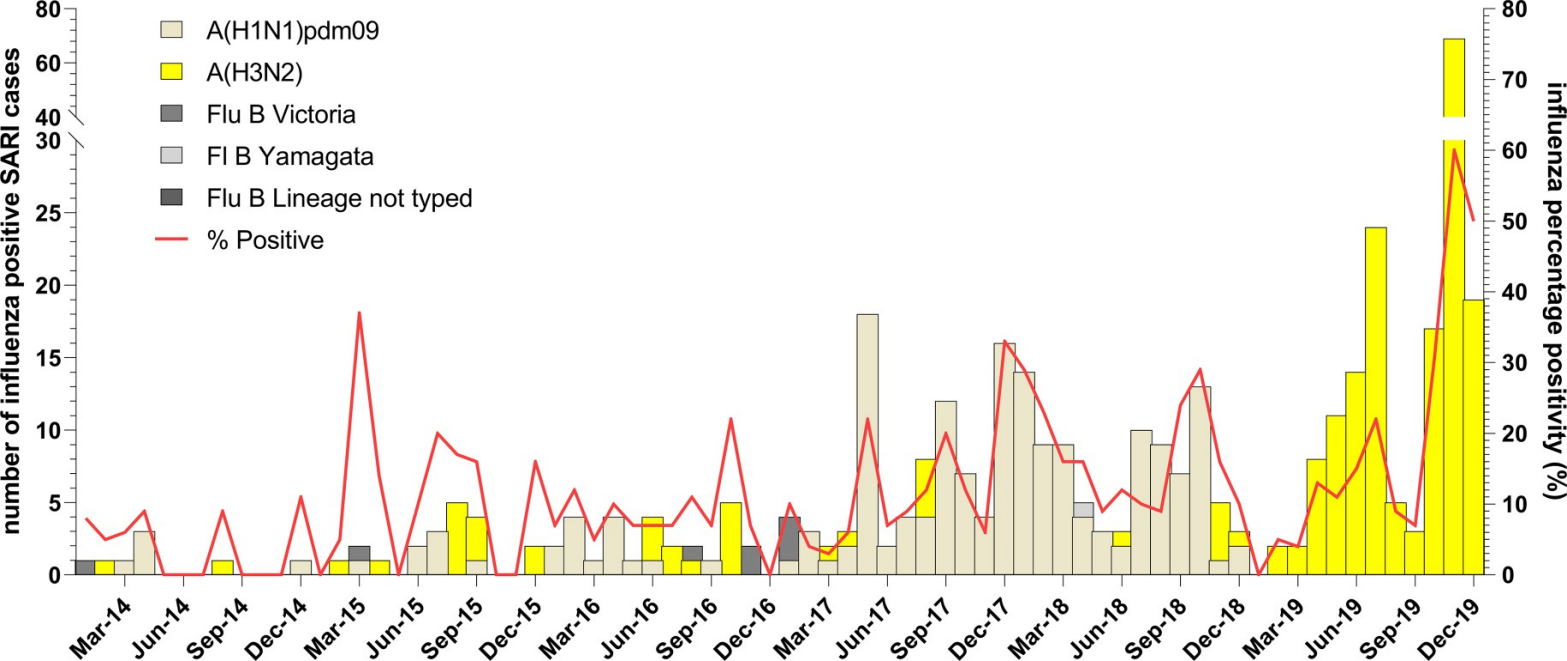
Distribution of influenza A subtypes and B lineages by months in Ghana from 2011 to 2019. Influenza positivity rate and the number of influenza A subtypes and B lineages was plotted against months for SARI.

The absolute number of SARI patients was greatest in those <5yo and lowest in those ≥ 65yo. A total of 535/3,429 (15.6%) SARI patients were positive for influenza with a positivity rate of 24% (107/450) occurring in the 5–14 year old group (See Fig 2). We received and tested more male SARI samples as compared to females but there was not a significant difference in rates of influenza positivity between genders (p-value = 0.454) (See Table 1). See S3 Table for influenza subtype variation with age. SARI cases were more prevalent during the wet seasons (n = 2060) than the dry seasons; however, influenza positive SARI was higher in the dry season (17.1% vs. 14.6%, p = 0.05) (See Table 1). The peak positivity rate of influenza-associated SARI cases were increasing yearly and peaked at 23.5% in 2019 (See Fig 5). For influenza positive SARI cases, A H1N1pdm09 and B Yamagata peaked in 2018 but were completely absent in 2019 (See Fig 4).

### Influenza seasonality

**Fig 6:**
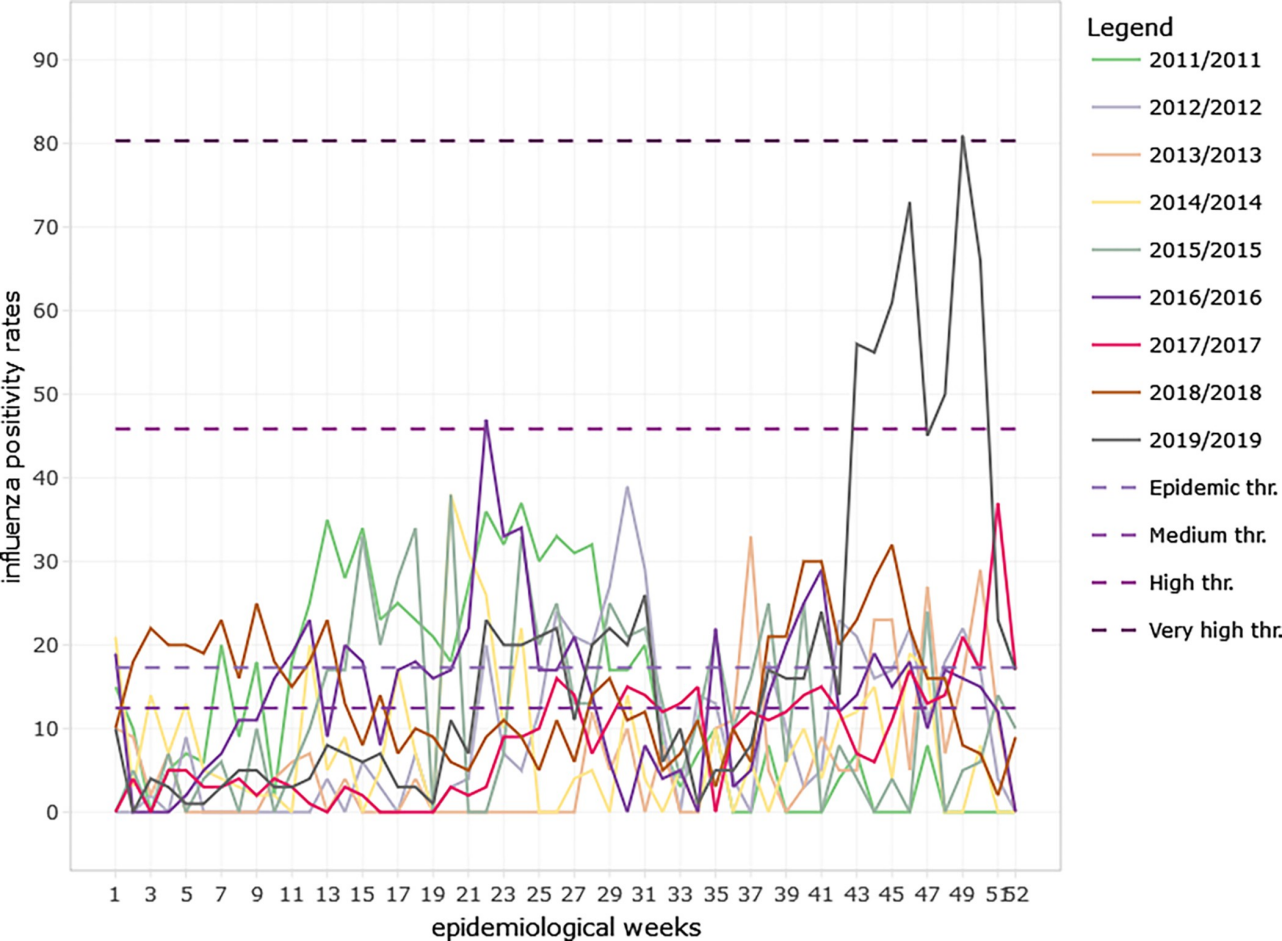
Influenza positivity rates among ILI and SARI cases in Ghana by epidemiological weeks from 2011 to 2019. Intensity thresholds were generated using the MEM web application (R version 4.2.1) and shown with dotted lines.

Influenza positivity rates varied significantly from 2011 to 2019 for both ILI (p<0.001) and SARI (p<0.001) cases (See Table 1). The influenza positivity rates for both ILI and SARI did not show a clear seasonal pattern over the 9-year period (See Figs 3 & 5). The average onset of the influenza epidemic season starts from week 22 (95% CI; 9, 38) towards the beginning of June. Throughout the 9-year period, influenza activity occurred all year round with low intensity on average except in 2019 when high influenza activity was recorded from week 43 to week 51 (See Fig 6) The high intensity during this period was mainly driven by influenza A H3N2 and influenza B Victoria lineage (See Figs 3 & 5).

## Discussion

Ghana has successfully established a virological surveillance system for influenza viruses. This system has the ability to rapidly detect circulating influenza viruses and provide timely information to the Global Influenza Surveillance and Response System (GISRS).

The observations for both ILI and SARI surveillance show that in Ghana, influenza is most prevalent in children 5–14 year old, which is the school going age. These findings are similar to prior estimates from a Ghanaian study [23]. Although this study did not explore the transmission of influenza by age group in a community setting, other studies have suggested that children and young adults that intersect appear to drive the spread of influenza in community settings [24, 25]. These age groups are potential target groups for future interventions that will help reduce the spread of influenza transmission. In addition, the lower influenza prevalence among the elderly may be attributable to the elderly not seeking care at the clinics or hospitals where surveillance sites are located. Instead, they often self-medicate or seek care at pharmacies. Therefore, it might be prudent to expand the surveillance sites outside of the hospital and clinic in order to accurately capture those who seek care elsewhere.

Historically, Ghana experiences influenza cases throughout the year, but has two expected peaks. The first occurs during the major rainy season (April to June) and the second in the minor rainy season (August to October), with resultant peaks in ILI and SARI [9]. However, between 2011 and 2018, the double peak pattern was not typically seen for patients with ILI. Rather, the temporal pattern of influenza cases varied significantly from year to year and included years with multiple peaks (2015) and years with only one peak (2011). Also, in several years (2011, 2012, 2015, 2017 and early 2018) the ILI peaks occurred prior to or between the rainy seasons. For SARI cases, the rates of influenza positive cases were more likely to follow the expected pattern with peaks in each of the rainy seasons, and this was clearly seen in 2014 through 2017. Factors impacting these observed patterns could include differences in awareness/screening or differences in symptom presentation, which may have impacted diagnosis. It is also possible that the year-to-year variation was impacted by humidity during the years of this study. This is an issue that should be assessed prospectively if humidity and rainfall data is available. Our data is in concordance with influenza data from other tropical countries where influenza circulation is year-round and patterns are difficult to predict [26]. Influenza positivity rates were higher in the dry season (November to March) compared to the wet seasons (April to October) although numerically more ILI/SARI samples were received at the NIC during the wet seasons. The high influenza positivity rates observed in the dry season was mainly driven by the 2019 influenza outbreak that occurred from September to December, 2019.

In more recent years, influenza positive SARI cases were present throughout the year. However, in 2014 and 2015 there were multiple months where surveillance did not detect any influenza positive SARI cases. This could be a result of less virulent influenza strains circulating in those years. Influenza was present in communities and caused ILI, but perhaps was not virulent enough to cause SARI. There is also the potential of sampling error and bias.

The distribution of influenza rates in Ghana over the 9-year period presents similar patterns as that of other West African countries (for example Cote D’Ivoire and Senegal) [27]. There are years where Ghana mirrors the global and/or West African peaks and lineages, but there are also many years with significant variability. For instance, in 2013, Influenza B predominated in West Africa, but very little Influenza B was seen in Ghana, and in 2014 Influenza A predominated globally, but Influenza B predominated for most of the year in Ghana [24]. This unpredictability limits the generalizability of global and West African data in predicting influenza trends. It however presents a unique opportunity for Ghana and other West African countries to submit samples and data to the WHO Collaborating Centres so that patterns can be better represented in biannual vaccine candidate strain selections. These observations also highlight the need for multiple surveillance sites and multiple pathogen detection.

Our study has a few limitations. The influenza positivity patterns seen in this study may be limited because the sentinel sites were required to send only the first five ILI samples for testing each week, although sometimes more samples are sent. This is because the study aimed to have a defined denominator, which is the first ILI cases seen at the health facilities in the given week. In addition, this study did not analyze for other respiratory viruses as the etiology of the ILI cases, and there is not sufficient data on seasonal variation of or weather influences on these viruses. Again, Ghana did not start SARI surveillance until 2014 due to constraints beyond the scope of this study. We however believe that these limitations did not affect our data adversely.

## Conclusions

This study presents 9 years of surveillance data from outpatient and inpatient settings on influenza activity as well as the influenza A subtypes and B lineages that drive the activity. The findings of the study provide useful information for influenza vaccine selection, as well as guide in developing guidelines in vaccine administration. There is a continued need for surveillance of influenza and other respiratory viruses linked to ILI and SARI to better understand patterns/variations and to better protect the public.

## Supporting information

S1 Table Bonferroni analysis. (DOCX)

S2 Table Influenza associated ILI and SARI by symptoms. (DOCX)

S3 Table A: Influenza A subtypes by year, season, and age. B: Influenza B lineages by year, season, and age.
(DOCX)

## References

[1] Gessner B, Shindo N, Briand S. Seasonal influenza epidemiology in sub-saharan africa: A systematic review. *Lancet Infectious Diseases*. 2011;11(3):223. http://search.ebscohost.com/login.aspx?direct=true&db=rzh&AN=104836175&site=ehost-live&scope=site. doi: 10.1016/S1473-3099(11)70008-1 21371656

[2] GBD 2017 Influenza Collaborators. Mortality, morbidity, and hospitalisations due to influenza lower respiratory tract infections, 2017: An analysis for the global burden of disease study 2017. *Lancet Respir Med*. 2019;7(1):69–89. S2213-2600(18)30496-X [pii]. doi: 10.1016/S2213-2600(18)30496-X 30553848 PMC6302221

[3] World Health Organization–influenza burden of disease. World Health Organization Web site [cited 2019 Jan 23]. Avaible from: https://www.who.int/influenza/surveillance_monitoring/bod/en/.

[4] Caini S, Spreeuwenberg P, Kusznierz G, Rudi J, Owen R, Pennington K, et al. Distribution of influenza virus types by age using case-based global surveillance data from twenty-nine countries, 1999–2014. *BMC Infect Dis*. 2018;18(1):269–y. doi: 10.1186/s12879-018-3181-y 29884140 PMC5994061

[5] Chaves S, Aragon D, Bennett N, Cooper T, D’Mello T, Farley M, et al. Patients hospitalized with laboratory-confirmed influenza during the 2010–2011 influenza season: Exploring disease severity by virus type and subtype. *The Journal of infectious diseases*. 2013 Oct 15;208(208). 1305–14. doi: 10.1093/infdis/jit316 [PubMed]. 23863950

[6] Patterson K. The influenza epidemic of 1918–1919 in the gold coast. *Transactions of the Historical Society of Ghana*. 1995(1):205–225. http://www.jstor.org/stable/41406618. doi: 10.1017/s0021853700028012 11675764

[7] Addy P, Mingle J, Nowacki W. The 1973 hong kong influenza epidemic in ghana. part II. virological and seroepidemiological data on hong kong influenza in ghana. *Ghana Medical Journal* 15(1). 1976;15(1):53–57.

[8] Mingle J, Oforo-Adjei E, Oforo-Adjei D, Sackey S. An outbreak of influenza A (H3N2) in Accra in 1996. *Ghana Medical Journal*. 1996 (Volume 31a):799–801.

[9] Bonney J, Kronmann K, Lindan CP, Asante I, Parbie P, Aboagye J, et al. Virological surveillance of influenza-like illness among children in ghana, 2008–2010. *The Journal of Infectious Diseases*, Volume 206, Issue suppl_1, 15 December 2012, Pages S108–S113, doi: 10.1093/infdis/jis577 23169955

[10] Hogan B, Ammer L, Zimmermann M, Binger T, Krumkamp R, Sarpong N, et al. Burden of influenza among hospitalized febrile children in Ghana. *Influenza Other Respiratory Viruses*. 2017;11(6):497–501. doi: 10.1111/irv.12507 28991406 PMC5705687

[11] Dawood F, Iuliano A, Reed C, Meltzer M, Shay D, Cheng P, et al. Estimated global mortality associated with the first 12 months of 2009 pandemic influenza A H1N1 virus circulation: A modelling study. *Lancet Infectious Disease*. 2012;12(9):687–695. doi: 10.1016/S1473-3099(12)70121-4 . 22738893

[12] Neumann G, Kawaoka Y. The first influenza pandemic of the new millennium. *Influenza Other Respiratory Viruses*. 2011 May;5(3):157–66. doi: 10.1111/j.1750-2659.2011.00231.x Epub 2011 Feb 28. ; PMCID: PMC3073629. 21477134 PMC3073629

[13] Viboud C, Simonsen L. Global mortality of 2009 pandemic influenza A H1N1. *Lancet Infectious Diseases*.2012;12(9):651. http://search.ebscohost.com/login.aspx?direct=true&db=asn&AN=79337968&site=ehost-live&scope=site. doi: 10.1016/S1473-3099(12)70152-4 22738892

[14] Steffen C, Debellut F, Gessner B, Kasolo F, Yahaya A, Ayebazibwe N, et al. Improving influenza surveillance in sub-saharan africa. World Health Organization Website. https://www.who.int/bulletin/volumes/90/4/11-098244/en/. Updated 2012. Accessed 28May, 2020. 10.2471/BLT.11.098244 PMC3324873 22511827

[15] Kronmann KC, Ampofo W, Nzussouo T, Wasfy MO, Agbenohevi P, Carroll J, et al. Building military influenza surveillance capacity in west africa. *Mil Med*. 2013;178(3):306–314. doi: 10.7205/MILMED-D-12-00273 23707118

[16] Ghana Health Services 2014 Annual Report. *Ghana Health Services*. 2015.

[17] Climates to travel. Climates To Travel—World Travel Guide Web site. [cited 2020 May 29]. Available from: https://www.climatestotravel.com/climate/ghana.

[18] Ghana—climate. Global Security—Military—Africa- Ghana—Climate Web site. [cited 2020 May 29]. Available from: https://www.globalsecurity.org/military/world/africa/gh-climate.html.

[19] Ghana statistical services. Ghana Statistical Services Web site. [cited 2020 May 29]. Available from: https://www.statsghana.gov.gh/nationalaccount_macros.php?Stats=MTA1NTY1NjgxLjUwNg==/webstats/s679n2sn87. Updated 2019.

[20] Protocol for human influenza surveillance in Ghana. Disease Surveillance Department. 2016. [cited 2020 May 24]. Available from: https://www.afro.who.int/publications/protocol-human-influenza-surveillance-ghana.

[21] CDC protocol of real-time RT-PCR for Influenza A(H1N1), WHO Collaborating Centre for Influenza at Centers for Disease Control, revision 2, 06October2009. [cited 2020 May 24]. Available from: https://www.who.int/csr/resources/publications/swineflu/CDCRealtimeRTPCR_SwineH1Assay-2009_20090430.pdf?ua=1.

[22] Manual for the laboratory diagnosis and virological surveillance of influenza. World Health Organization Global Influenza Surveillance Network. 2011… [cited 2020 May 24]. Available from: https://apps.who.int/iris/handle/10665/44518.

[23] Ntiri M, Duque J, McMorrow M, Frimpong J, Parbie P, Badji E, et al. Incidence of medically attended influenza among residents of Shai-Osudoku and Ningo-Prampram Districts, Ghana, May 2013—April 2015 (2016). *BMC Infect Dis*.;16(1):757. 10.1186/s12879-016-2078-x PMC5155389 27964716

[24] Yang L, Chan K, Suen L, Chan K, Wang X, Cao P, et al. Impact of the 2009 H1N1 Pandemic on Age-Specific Epidemic Curves of Other Respiratory Viruses: A Comparison of Pre-Pandemic, Pandemic and Post-Pandemic Periods in a Subtropical City (2015). Goldstein E, editor. *PLoS One*.;10(4):e0125447. 10.1371/journal.pone.0125447 PMC4416050 25928217

[25] Choe Y, Smit M, Mermel L. Comparison of Common Respiratory Virus Peak Incidence Among Varying Age Groups in Rhode Island, 2012–2016 (2020). *JAMA Netw open*.1;3(5):e207041. 10.1001/jamanetworkopen.2020.7041 PMC7221508 32401314

[26] Hirve S, Newman LP, Paget J, Azziz-Baumgartner E, Fitzner J, et al. (2016) Influenza Seasonality in the Tropics and Subtropics–When to Vaccinate?. *PLOS ONE* 11(4): e0153003. doi: 10.1371/journal.pone.0153003 27119988 PMC4847850

[27] World Health Organization—FluNet. World Health Organization. [cited 2020 May 24]. Available from: https://www.who.int/influenza/gisrs_laboratory/flunet/en/.

