# Supplementary material for: Epidemiology of influenza in Ghana, 2011 to 2019
Source: PLOS Glob Public Health. 2022 Dec 9;2(12):e0001104. doi: 10.1371/journal.pgph.0001104 (PMC10021352; doi:10.1371/journal.pgph.0001104)
Supplement: S1 Table — (DOCX) [file pgph.0001104.s001.docx]

**S1 Table - Bonferroni Analysis**

|  | **ILI** | | | | | |  | **SARI** | | | | | | |
| --- | --- | --- | --- | --- | --- | --- | --- | --- | --- | --- | --- | --- | --- | --- |
| **Age group** | **Comparator** | **Contrast** | **Std** | **Z** | **P>│Z│** | **Bonferroni** | | **Contrast** | **Std** | **Z** | **P>│Z│** | **Bonferroni** | |  |
|  | **group** |  | **Err** |  |  | **95%CI** | |  | **Err** |  |  | **95%CI** | |  |
| 5-14years | <5years | 0.47 | 0.057 | 8.21 | 0 | 3.02 | 0.638 | 0.547 | 0.132 | 4.12 | 0 | 0.157 | 0.937 |  |
| 15-24years |  | 0.258 | 0.059 | 4.39 | 0 | 0.085 | 0.043 | 0.552 | 0.143 | 3.85 | 0 | 0.131 | 0.975 |  |
| 25-44years |  | -0.277 | 0.057 | -4.86 | 0 | -0.444 | -0.109 | -0.176 | 0.145 | -1.21 | 0.227 | -0.602 | 0.251 |  |
| 45-64years |  | -0.603 | 0.082 | -7.28 | 0 | -0.847 | -0.359 | -0.662 | 0.194 | -3.42 | 0.001 | -1.23 | -0.093 |  |
| ≥65years |  | -0.6 | 0.134 | -4.47 | 0 | -0.994 | -0.206 | -0.933 | 0.296 | -3.15 | 0.002 | -1.802 | -0.063 |  |
| 15-24years | 5-14years | -0.212 | 0.061 | -3.46 | 0.001 | -0.393 | -0.032 | 0.006 | 0.166 | 0.04 | 0.971 | -0.481 | 0.493 |  |
| 25-44years |  | -0.747 | 0.06 | -12.5 | 0 | -0.923 | -0.571 | -0.723 | 0.167 | -4.32 | 0 | -1.214 | -0.231 |  |
| 45-64years |  | -1.073 | 0.085 | -12.66 | 0 | -1.322 | -0.825 | -1.209 | 0.211 | -5.74 | 0 | -1.827 | -0.59 |  |
| ≥65years |  | -1070 | 0.135 | -7.91 | 0 | -1.467 | -0.673 | -1.48 | 0.308 | -4.81 | 0 | -2.382 | -0.577 |  |
| 25-44years | 15-24years | -0.535 | 0.061 | -8.74 | 0 | -0.715 | -0.355 | -0.729 | 0.176 | -4.14 | 0 | -1.246 | -0.211 |  |
| 45-64years |  | -0.861 | 0.086 | -10.03 | 0 | -1.112 | -0.609 | -1.214 | 0.218 | -5.58 | 0 | -1.854 | -0.58 |  |
| ≥65years |  | -0.858 | 0.136 | -6.31 | 0 | -1.256 | -0.459 | -1.486 | 0.313 | -4.75 | 0 | -2.403 | -0.568 |  |
| 45-64years | 25-45years | -0.326 | 0.084 | -3.85 | 0 | -0.575 | -0.074 | -0.486 | 0.219 | -2.22 | 0.026 | -1.129 | 0.157 |  |
| ≥65years |  | -0.322 | 0.135 | -2.39 | 0.017 | -0.72 | 0.074 | -0.757 | 0.313 | -2.42 | 0.016 | -1.677 | 0.163 |  |
| ≥65years | 45-64years | 0.003 | 0.148 | 0.02 | 0.982 | -0.431 | 0.437 | -0.271 | 0.338 | -0.8 | 0.423 | -1.264 | 0.722 |  |

Note: Any p-value $\geq$ 0.0033 is not statistically significant
