# Supplementary material for: Epidemiology of influenza in Ghana, 2011 to 2019
Source: PLOS Glob Public Health. 2022 Dec 9;2(12):e0001104. doi: 10.1371/journal.pgph.0001104 (PMC10021352; doi:10.1371/journal.pgph.0001104)
Supplement: S2 Table — (DOCX) [file pgph.0001104.s002.docx]

**S2 Table - Influenza associated ILI and SARI by symptoms**

|  | **Headache** | **Myalgia** | **Sore Throat** | **Coryza** |
| --- | --- | --- | --- | --- |
|  | **ILI** | | | |
| Odds Ratio (95%CI) | 1.1 (0.9-1.3) | 1.8 (1.2-2.5) | 1.0 (0.9-1.1) | 0.8 (0.6-1.1) |
|  | **SARI** | | | |
| Odds Ratio (95%CI) | 2.1 (1.6-2.7) | 0.9 (0.4-2.1) | 1.2 (1.1-1.4) | 1.4 (0.8-2.7) |
