# Supplementary material for: Epidemiology of influenza in Ghana, 2011 to 2019
Source: PLOS Glob Public Health. 2022 Dec 9;2(12):e0001104. doi: 10.1371/journal.pgph.0001104 (PMC10021352; doi:10.1371/journal.pgph.0001104)
Supplement: S3 Table — B: Influenza B lineages by year, season, and age. (DOCX) [file pgph.0001104.s003.docx]

**Table A: Influenza A subtypes by year, season, and age**

|  | **ILI** | | | **SARI** | | |
| --- | --- | --- | --- | --- | --- | --- |
|  | **A(H3N2)**  **n(%)** | **A(H1N1)pdm09**  **n(%)** | **p-value** | **A(H3N2)**  **n(%)** | **A(H1N1)pdm09**  **n(%)** | **p-value** |
| Year |  |  | <0.001 |  |  |  |
| 2011 | 42(25.0) | 123(75.0) |  |  |  |  |
| 2012 | 47(39.0) | 72(61.0) |  |  |  |  |
| 2013 | 29(76.0) | 9(24.0) |  |  |  |  |
| 2014 | 33(72.0) | 13(28.0) |  | 32 | 5(71.0) | <0.001 |
| 2015 | 40(52.0) | 37(48.0) |  | 14(67.0) | 7(33.0) |  |
| 2016 | 143(74.0) | 49(26.0) |  | 16(53.0) | 14(47.0) |  |
| 2017 | 106(34.0) | 207(66.0) |  | 27(27.0) | 74(73.0) |  |
| 2018 | 66(19.5) | 273(80.5) |  | 15(15.3) | 83(84.7) |  |
| 2019 | 787(99.6) | 3(0.4) |  | 174(100) | 0(0.0) |  |
| **Season** |  |  | 0.730 |  |  | 0.129 |
| Wet | 694(61.9) | 428(38.2) |  | 135(54.4) | 113(45.6) |  |
| Dry | 599(62.6) | 358(37.4) |  | 113(61.8) | 70(38.3) |  |
| **Age** |  |  | 0.024 |  |  | 0.022 |
| <5years | 316(57.8) | 231(42.2) |  | 89(50.9) | 86(49.1) |  |
| 5-14years | 262(61.4) | 165(38.6) |  | 54(69.2) | 24(30.8) |  |
| 15-24years | 301(68.3) | 140(31.8) |  | 40(52.0) | 37(48.1) |  |
| 25-44years | 268(60.4) | 176(39.6) |  | 42(71.2) | 17(28.8) |  |
| 45-64years | 91(63.6) | 52(36.4) |  | 16(55.2) | 13(44.8) |  |
| 65years and above | 30(68.2) | 14(31.8) |  | 7(58.3) | 5(41.7) |  |

**Table B: Influenza B lineages by year, season, and age**

|  | **ILI** | | | **SARI** | | |
| --- | --- | --- | --- | --- | --- | --- |
|  | **Victoria n (%)** | **Yamagata**  **n (%)** | **p-value** | **Victoria n(%)** | **Yamagata n(%)** | **p-value** |
| **Year** |  |  | <0.001 |  |  |  |
| 2011 | 25(100.0) | 0(0.0) |  |  |  |  |
| 2012 | 0(0.0) | 0(0.0) |  |  |  |  |
| 2013 | 1(100.0) | 0(0.0) |  |  |  |  |
| 2014 | 46(98.0) | 1(2.0) |  | 2(100.0) | 0(0.0) | <0.001 |
| 2015 | 3(8.0) | 35(92.0) |  | 2(67.0) | 1(33.0) |  |
| 2016 | 80(66.7) | 39(33.3) |  | 9(75.0) | 3(25.0) |  |
| 2017 | 11(28.2) | 28(71.8) |  | 8(57.1) | 6(42.9) |  |
| 2018 | 112(39.9) | 169(60.1) |  | 12(30.0) | 28(70.0) |  |
| 2019 | 183(100.0) | 0(0.0) |  | 32(100.0) | 0(0.0) |  |
| **Season** |  |  | 0.010 |  |  | 0.739 |
| Wet | 271(68.3) | 126(31.7) |  | 32(61.5) | 20(38.5) |  |
| Dry | 190(56.4) | 147(43.6) |  | 33(64.7) | 18(35.3) |  |
| **Age** |  |  | 0.056 |  |  | 0.327 |
| <5years | 128(64.0) | 72(36.0) |  | 31(70.5) | 13(29.6) |  |
| 5-14years | 141(65.0) | 76(35.0) |  | 14(48.3) | 15(51.7) |  |
| 15-24years | 84(66.1) | 43(33.9) |  | 7(77.8) | 2(22.2) |  |
| 25-44years | 76(60.8) | 49(39.2) |  | 9(64.3) | 5(35.7) |  |
| 45-64years | 16(41.0) | 23(59.0) |  | 3(60.0) | 2(40.0) |  |
| 65years and above | 6(46.2) | 7(53.9) |  | 0(0.0) | 0(0.0) |  |
